# A Rare and Lethal Complication: Cerebral Edema in the Adult Patient with Diabetic Ketoacidosis

**DOI:** 10.1155/2018/5043752

**Published:** 2018-03-21

**Authors:** Christopher W. Meaden, Beth J. Kushner, Stacey Barnes

**Affiliations:** Emergency Department, St. Joseph's Regional Medical Center, 703 Main Street, Paterson, NJ 07503, USA

## Abstract

Commonly seen in the emergency department, diabetic ketoacidosis is a potentially lethal sequela of uncontrolled diabetes mellitus. In the adult population, a rare complication of diabetic ketoacidosis is cerebral edema. This case report discusses a 26-year-old male with new onset diabetes mellitus who developed cerebral edema leading to death.

## 1. Introduction

An unfortunate, yet common, complication of uncontrolled diabetes is diabetic ketoacidosis (DKA). A combination of elevated blood glucose in the setting of depleted insulin availability creates an acidic environment from the production of *β*-hydroxybutyric acid and acetoacetic acid [1]. DKA has been linked to several devastating metabolic derangements including osmotic diuresis, depletion of intracellular potassium, accumulation of toxic ketoacids, and dysregulation of sodium hydrogen exchanger mechanisms [2, 3]. A destructive consequence secondary to these abnormalities is cerebral edema (CE) that has been documented throughout the pediatric literature, but more sporadically in the adult literature. Cerebral edema results in poor outcomes with mortality occurring in 21–25% of patients and neurological morbidity occurring in 15–26% of patients [4].

## 2. Case Report

A 26-year-old African American male, with no known past medical or surgical history, presented with increased urinary frequency that was ongoing for ten days and associated with right-sided abdominal pain, polydipsia, nausea, vomiting, and diarrhea. Aside from a recent trip to Jamaica, the remainder of his history was negative for significant findings.

On initial presentation, the patient appeared uncomfortable, had dry oral mucosa, and was awake, alert, and oriented to person, place, and time. His pulse was 119 beats per minute, blood pressure was 147/83 mmHg, respiratory rate was 18 breaths per minute, pulse oximetry oxygen-hemoglobin saturation was 98 percent on room air, and his temperature was 97.8 degrees Fahrenheit. The patient was tachycardic with a regular rhythm and without murmur on cardiac auscultation, his lungs were clear to auscultation bilaterally without rales, rhonchi, or wheezing, and his abdomen was soft, nontender, and nondistended.

Promptly after bedside evaluation, a bedside finger stick blood glucose test was performed giving a value of greater than 650 mg/dl (this was the maximum point of care value). The patient was significantly dehydrated leading to poor peripheral venous access; therefore, an arterial blood gas (ABG) was performed on the patient to assess acid-base status and electrolytes, while venous access was established. The results of the ABG performed on room air, were pH 7.14, PO_2_ 114 mmHg, and PCO_2_ 17 mmHg. Initially oral hydration, with ice water provided by nursing staff, was started, while a femoral triple lumen central venous catheter was placed and 10 units of regular human insulin was given subcutaneously. The placement of the central venous catheter and administration of the subcutaneous insulin occurred approximately one hour after initial evaluation and measurement of blood glucose level with glucometer. Upon placement of the central venous catheter, the patient was given two liters of normal saline, over one hour. Venous blood was drawn from the central venous catheter at the time of placement and sent for laboratory analysis. The patient's initial lab values sent 1 hour from initial evaluation are provided on Table 1. 30 minutes after the results of the initial labs were obtained, an insulin drip was started at an initial rate of 12 units per hour (0.1 U/kg), as he weighed 122 kilograms. Chest X-ray was performed to rule out other pathology and was noted to be normal. EKG performed on the patient revealed sinus tachycardia with peaked T waves; the patient was placed on a cardiac monitor for these changes and continued to receive the insulin drip. One gram of ceftriaxone was administered for empiric antibiotic coverage, as coexisting sepsis infection had not yet been ruled out. The patient was subsequently accepted for admission to the medical intensive care unit (ICU) for new onset diabetes mellitus with diabetic ketoacidosis, his insulin drip was continued, and he was started on continuous intravenous fluids of 0.9% normal saline at a rate of 200 cc/hour.

Two hours after beginning the insulin drip, the repeat point of care blood glucose was 446 mg/dl. Subsequently, the insulin drip was decreased from 12  units/hour to 6 units/hour so that the serum blood glucose would not be lowered too rapidly. At this time, the patient began to complain of a diffuse frontal headache, which was treated with 2 mg of morphine and a noncontrast computed tomography (CT) of the head was ordered.

**Figure 1:**
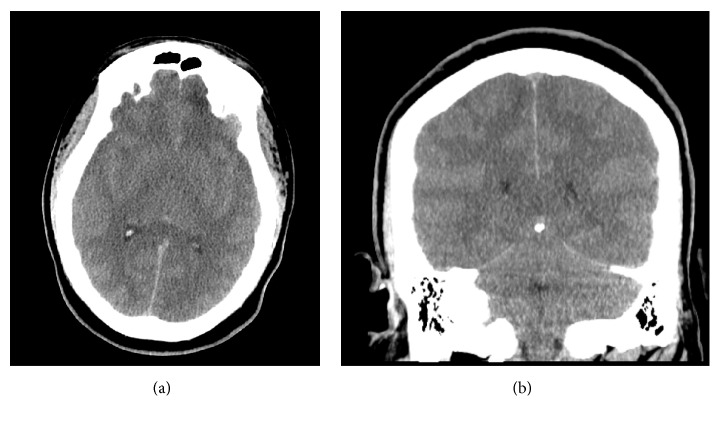
Axial cut (a) and coronal cut (b) of the noncontrast computed tomography (CT) of the head of the patient after becoming obtunded with nonreactive pupils. The images show diffuse cerebral edema with compression of lateral ventricles, effacement of the sulci, and obliteration of the perimesencephalic cisterns.

After his headache developed, the patient became progressively more lethargic. One hour later, while awaiting CT, he became unresponsive and had fixed and dilated pupils on exam. He was subsequently intubated for airway protection and respiratory support. The previously ordered CT of the head without contrast was completed approximately 30 minutes after intubation. Repeat labs were performed at this time, as well (Table 1). The noncontrast CT of the head revealed severe diffuse brain swelling and obliteration of the sulci and ventricles and perimesencephalic cisterns, as read by the radiology department (Figure 1).

Neurosurgery was consulted and the decision was made to transfer the patient to the surgical ICU for intracranial pressure (ICP) monitor placement and continued management, as there was concern for impending herniation. He was given an 80 cc 23% hypertonic saline bolus and then placed on a 3% NaCl drip at an initial rate of 75 cc/hour. The goal plasma sodium level was 150–155 mEq/L. After placement of ICP monitor, initial pressure was measured to be 35–38 mmHg and 40 G of mannitol was given intravenously; no improvement in patient's neurological status was noted. Throughout the day, the patient's clinical condition remained unchanged.

The following morning, 22 hours after the initial CT head without contrast, the patient was noted to be in cardiac arrest with pulseless electrical activity as the initial rhythm. He was resuscitated at bedside using standard advanced cardiovascular life support (ACLS). Repeat labs drawn immediately after return of spontaneous circulation were drawn and are shown in Table 1. Five hours after the first cardiac arrest, the patient again lost pulses and was pronounced dead after a repeated attempt at resuscitation was unsuccessful. An overview of critical events of the patient's course is shown in Table 2.

## 3. Discussion

Published rates reporting the mortality of CE associated with DKA range from 21 to 35%, with 35% as reported by the British Pediatric Surveillance Unit [5]. A combined mechanism of vasogenic and cellular causes is thought to cause the significant CE. Not only does DKA play a role in creating CE, but also it appears that CE is often made worse with inappropriate and sometimes overaggressive therapies. Noted throughout case reports are patients who present at younger ages and have longer duration of symptoms. These patients were more acidotic, dehydrated, and hyperglycemic, which appear to increase the risk for development of CE [4]. Treatment strategies, including bolus administration of insulin and bicarbonate, appear to play a significant role in development of CE as well. Administration of medications that depress respiratory drive, such as morphine, can decrease a protective hyperventilation that could further worsen an acidotic state; these medications and standard mechanical ventilation settings in patients who are intubated should be avoided [3, 6].

Longitudinal prospective case controlled studies, like the one done by Lawrence et al., were performed to identify various risk factors for patients who could develop CE. This study identified that younger patients, with a mean age of 4.5 years and an age range of 1.1 to 15.3 years, were significantly more impacted by CE. However, Troy et al. (2005), and other case reports, show new onset adult diabetes presenting with DKA complicated by CE [7]. Another trend noted in the Lawrence et al. case review is the development of CE after treatment, with a median time of 5.8 hours. The timing of CE onset after treatment suggests CE is a complication which must be recognized by emergency department staff. Other lab abnormalities that were noted amongst patients with CE, but not found to be statistically significant, include lower pH, decreased sodium bicarbonate, elevated blood urea nitrogen (BUN), and increased hyperglycemia [4].

Many different mechanisms have been proposed on a cellular level to explain CE. Prior to the onset of treatment with insulin, free water consumption results in water moving from the intravascular space and into the CNS across the blood brain barrier which causes brain swelling. This water transfer can even occur in a nonhypertonic state [8]. In a juvenile mouse model, created to explain how treatments can cause CE on a cellular level, it was found that mice which received combined bicarbonate and insulin therapy developed perineuronal and perivascular edema with microvacuolation of the white matter tracts [9].

A review of 26 case subjects (aged 1–15 years) with CE, completed by Muir et al., found CE to be in a bimodal temporal relationship; older children presented earlier within 0–6 hours after starting treatment, while younger patients presented with median time of 14.3 hours after starting treatment. Early acknowledgement of increased intracerebral pressure can lead to treatment strategies such as elevation of HOB to 30 degrees, administration of mannitol at dosages as high as 1 G/kg, and endotracheal intubation earlier in the treatment coarse. Treatment strategies to help prevent CE should include gradual rehydration over 48 hours, avoidance of boluses of sodium bicarbonate and 0.9% NaCl (normal saline), and serial neurologic examinations. These strategies should occur in a protocol based fashion to help catch early signs of neurologic impairment [10].

## 4. Conclusion

In the emergency department, DKA is a commonly observed entity that is often treated according to algorithms which may lead to severe clinical consequences that are often underappreciated. Certain maneuvers such as moderately aggressive fluid hydration, frequent monitoring of sodium levels, and serum osmolality can help to prevent cerebral edema. As patients with early intervention are found to have the best outcomes, it is imperative to act on clinical indicators and recognize patients transitioning from subclinical to clinically significant cerebral edema.

## Figures and Tables

**Table 1:** Lab values during the patient's clinical course. Timing is shown from time of initial evaluation and blood glucose reading from handheld glucometer.

	1 hour	6 hours	28 hours
	(Initial labs)	(Unresponsive)	(Cardiac arrest)
Sodium (mEq/L)	132	139	170
Chloride (mEq/L)	92	100	138
Potassium (mEq/L)	6.4	3.8	3.7
Bicarbonate (mEq/L)	7	8	18
Calculated plasma Osmolarity (mEq/L of Water)^*∗*^	325	314	363
Blood urea nitrogen (mg/dl)	42	30	29
Creatinine (mg/dl)	2.0	1.42	3.78
Glucose (mg/dl)	838	471	243
Calculated anion gap	33	31	14
Acetone	“LARGE”		

^*∗*^Plasma osmolarity calculated as 2[Na] + [glucose]/18 + [BUN]/2.8.

**Table 2:** Timeline of patient course and critical events. Cerebral edema likely began to develop at hour 4.5 when patient began to complain of headache.

Time (hours)	Event
0	Initial evaluation and elevated glucose on finger stick glucometer
1	Femoral central venous catheter placed, 2 L 0.9% normal saline started, initial labs drawn and sent for analysis
2	Initial labs result
2.5	Insulin drip started at dose of 0.1 U/kg
4.5	Insulin drip dose reduced, patient complains of headache
5.5	Patient becomes unresponsive and is intubated
6	CT head without contrast shows cerebral edema
28	Patient has first cardiac arrest with successful return of spontaneous circulation
33	Patient expires

## References

[1] Chiasson J.-L., Aris-Jilwan N., Bélanger R. (2003). Diagnosis and treatment of diabetic ketoacidosis and the hyperglycemic hyperosmolar state. *Canadian Medical Association Journal*.

[2] Kitabchi A. E., Wall B. M. (1995). Diabetic ketoacidosis. *Medical Clinics of North America*.

[3] Rose K. L., Watson A. J., Drysdale T. A. (2015). Simulated diabetic ketoacidosis therapy in vitro elicits brain cell swelling via sodium-hydrogen exchange and anion transport. *American Journal of Physiology-Endocrinology and Metabolism*.

[4] Lawrence S. E., Cummings E. A., Gaboury I., Daneman D. (2005). Population-based study of incidence and risk factors for cerebral edema in pediatric diabetic ketoacidosis. *Journal of Pediatrics*.

[5] Edge J. A., Hawkins M. M., Winter D. L., Dunger D. B. (2001). The risk and outcome of cerebral oedema developing during diabetic ketoacidosis.. *Archives of Disease in Childhood*.

[6] Tasker R. C., Lutman D., Peters M. J. (2005). Hyperventilation in sever diabetic ketoacidosis. *Pediatric Critical Care Medicine*.

[7] Troy P. J., Clark R. P., Kakarala S. G., Burns J., Silverman I. E., Shore E. (2005). Cerebral edema during treatment of diabetic ketoacidosis in an adult with new onset diabetes. *Neurocritical Care*.

[8] Fiordalisi I., Harris G. D., Gilliland M. G. F. (2002). Prehospital cardiac arrest in diabetic ketoacidemia - Why brain swelling may lead to death before treatment. *Journal of Diabetes and its Complications*.

[9] Rose K. L., Pin C. L., Wang R., Fraser D. D. (2007). Combined Insulin and Bicarbonate Therapy Elicits Cerebral Edema in a Juvenile Mouse Model of Diabetic Ketoacidosis. *Pediatric Research*.

[10] Muir A. B., Quisling R. G., Yang M. C. K., Rosenbloom A. L. (2004). Cerebral edema in childhood diabetic ketoacidosis: Natural history, radiographic findings, and early identification. *Diabetes Care*.

